# Elevated serum Cripto‐1 and VEGF levels in patients with non‐small cell lung cancer

**DOI:** 10.1096/fba.2022-00002

**Published:** 2022-05-03

**Authors:** Chunhua Xu, Jue Zou, Li Li, Qi Yuan, Wei Wang

**Affiliations:** ^1^ Department of Respiratory Medicine, Affiliated Nanjing Brain Hospital Nanjing Medical University Nanjing China; ^2^ Clinical Center of Nanjing Respiratory Diseases and Imaging Nanjing China; ^3^ Department of Pathology, Affiliated Nanjing Brain Hospital Nanjing Medical University Nanjing China

**Keywords:** biomarker, Cripto‐1, NSCLC, survival, VEGF

## Abstract

Cripto‐1 (CR‐1) facilitates vascular endothelial growth factor (VEGF) expression, and these markers are associated with various tumor cell proliferation, angiogenesis, and metastasis. The main aim of our study was to investigate the clinical value of CR‐1 and VEGF for non‐small cell lung cancer (NSCLC) patients. Serum samples were collected from 312 patients with NSCLC and 120 healthy controls. The levels of CR‐1 and VEGF were measured by enzyme‐linked immunosorbent assay (ELISA). The serum levels of CR‐1 and VEGF in NSCLC patients were significantly higher than those of healthy controls (*p* < 0.05). Elevated CR‐1 levels were associated with progression of NSCLC stage and higher CR‐1 was detected more in patients with distant metastasis (*p* < 0.05). Patients exhibiting low levels of serum CR‐1 had better overall survival than those with high levels (*p* < 0.05). The CR‐1 levels of NSCLC patients with postoperative recurrent were higher than those of nonrecurrent NSCLC patients. Our study suggests that serum CR‐1 and VEGF are useful biomarker for NSCLC patients.

**Abbreviations**
ADC: adenocarcinoma
AUC: area under the curve
CR‐1: Cripto‐1
ELISA: enzyme‐linked immunosorbent assay
NSCLC: non‐small cell lung cancer
OS: overall survival
PFS: progression‐free survival
ROC: receiver operating characteristic
SCC: squamous cell carcinoma
TNM: tumor node metastasis.
VEGF: vascular endothelial growth factor

## 1 INTRODUCTION

Lung cancer is one of the most fatal malignancies worldwide.^1^ Although the development of noninvasive approaches has improved the ability of early diagnosis of lung cancer, approximately 75% of patients are diagnosed at advanced lung cancer, leaving them with few effective treatments and a low 5 years survival rate.^2^, ^3^ The main cause of poor prognosis is deficient in effective methods for early diagnosis.

CR‐1, an epidermal growth factor‐cripto/FRL1/cryptic family protein, is closely related to early embryonic development.^4^, ^5^ Although it has been recognized as a biomarker for embryonic stem cell in human tissue, CR‐1 was overexpressed in various tumors, such as breast, lung, pancreatic, and ovarian cancers.^6^, ^7^ In recent years, CR‐1 has been shown an early tumor marker for diagnosis of breast and colon cancer.^8^

CR‐1 is an essential process in cancer growth, maintenance, and metastasis. It is controlled by the interplay of numerous proangiogenic factors including many growth factors, proteases, and cytokines. Of these, VEGF is the most extensively studied and is significantly related to tumor progression, metastasis, and prognosis. CR‐1 protein was highly expressed in lung cancer tissue and serum, and its expression level is related to prognosis.^9^, ^10^ VEGF can promote the development of tumors by providing nutrients for the growth of tumor cells.^11^ Previous studies found that VEGF is tightly correlated with low survival rate and poor prognosis of lung cancer patients.^12^ In this study, the levels of CR‐1 and VEGF in peripheral blood of lung cancer were determined by ELISA, and the relationship between the CR‐1 and VEGF and clinical significance was evaluated.

## 2 MATERIALS AND METHODS

### 2.1 Patients

This was a prospective, randomized, and controlled trial. From December 2016 to November 2019, we recruited 312 NSCLC patients for this study. Among patients with lung cancer, 94 had squamous cell carcinoma (SCC) and 218 had adenocarcinoma (ADC), 40 patients were stage I, 36 patients were stage II, 134 patients were stage III (stage IIIA = 60, stage IIIB = 30, stage IIIC = 44), and 102 patients were stage IV. As control subjects, 120 healthy volunteers were enrolled in the same period. Baseline demographic data are presented in Table 1. For all patients diagnosed as primary NSCLC by pathologist, in the light of the 2015 World Health Organization classification of lung tumors, and staged in the light of the 8th edition of the tumor‐node‐metastasis classification.^13^ The procedure included chest computed tomography scans, abdominal ultrasound, brain magnetic resonance imaging, and bone scans. The median follow‐up period was 24 months (range, 3–81 months). The follow‐up deadline was January 2020. Progression‐free survival (PFS) was defined as the time interval between the date of diagnosis and the date of recurrence. Overall survival (OS) was defined as the time interval between the date of diagnosis and the date of death or the last follow‐up.

**TABLE 1:** Clinical characteristics of NSCLC patients and healthy controls

Variables	NSCLC patients (*n* = 312)	Healthy control (*n* = 120)
Age (years)	68.7 ± 12.6	67.9 ± 12.7
Gender (*n*, %)		
Male	140 (44.9)	50 (41.7)
Female	172 (55.1)	70 (58.3)
Histology		
ADC	218(69.9)	
SCC	94 (30.1)	
TNM stage		
I + II	76 (24.4)	
III + IV	236 (75.6)	
Differentiation		
Well‐moderate	208 (66.7)	
Poor	104 (33.3)	
Lymph node metastases		
Absent	116 (37.2)	
Present	196 (62.8)	
Distant metastases		
Absent	210 (67.3)	
Present	102 (32.7)	
CR‐1 (ng/ml)	4.13 ± 1.38	1.03 ± 0.36
VEGF (pg/ml)	512.26 ± 110.58	388.56 ± 123.67

Abbreviations: ADC, Adenocarcinoma; SCC, Squamous cell carcinoma.

This study was approved by the Ethics Committee of Nanjing Chest Hospital and was carried out in accordance with national law and the current revised Declaration of Helsinki. Informed consent was obtained from all participants in the study.

### 2.2 Measurement of CR‐1 and VEGF Levels

Blood samples of NSCLC patients were obtained from each individual before therapy was initiated. The sample was centrifuged for 10 min at −4°C at 1500 × g. Supernatants were stored at −80°C to assess CR‐1 and VEGF levels. CR‐1 and VEGF levels were measured by ELISA kits (Quantikine; R&D Systems) according to the manufacturer's instructions. All tests are in duplicate, diluted appropriately, and the technicians were blinded to clinical data.

### 2.3 Statistical analysis

Statistical software (SPSS for Windows, version 20) was used for the analysis. All values are given as mean ± SD except for the survival period in which the mean ± SE was used. The values did not fit a standard distribution so nonparametric analysis was performed. The Mann–Whitney *U* test was used to compare patients and control groups, and the Kruskal–Wallis test was used to compare several groups. Spearman correlation of rank coefficient was used to analyze correlations between parameters. The cutoff value of the serum concentrations of parameters was calculated using a receiver operating characteristic (ROC) curve. Univariate survival analysis was performed using the Kaplan–Meier method and the log‐rank test. Multivariate analysis was conducted to determine an independent impact on survival using the Cox proportional hazard method. *p* < 0.05 was considered statistically significant.

## 3 RESULTS

### 3.1 Elevated serum CR‐1 and VEGF levels in NSCLC patients

**FIGURE 1:**
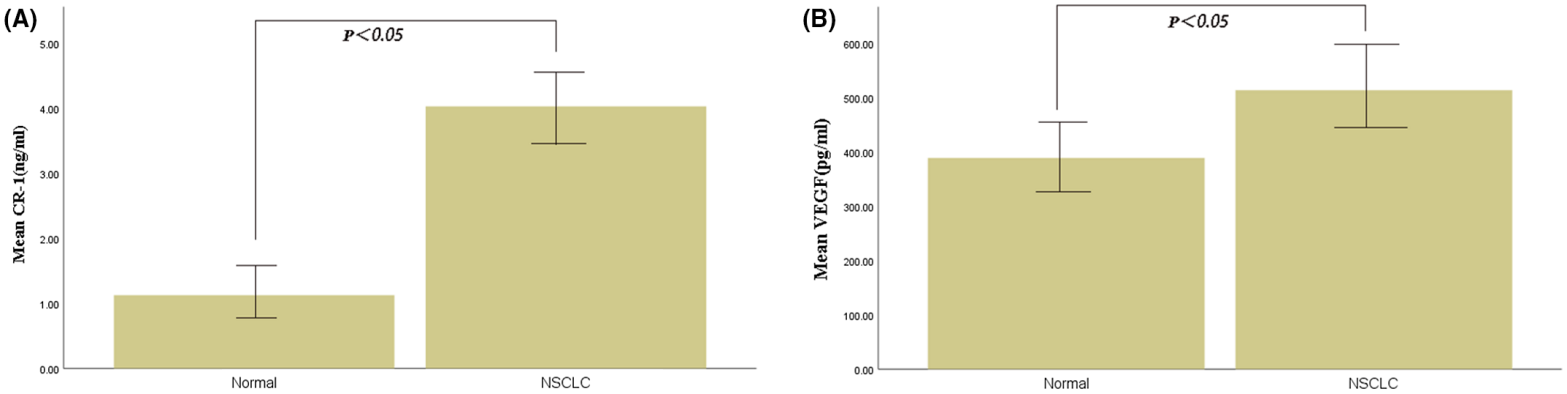
Serum CR‐1 (A) and VEGF (B) levels in NSCLC patients and control subjects

Serum CR‐1 levels in NSCLC patients were higher than those of healthy controls (4.13 ± 1.38 ng/ml vs. 1.03 ± 0.36 ng/ml, *p* < 0.05, Figure 1A, Table 1). The levels of serum VEGF in patients with NSCLC was also higher than those of healthy controls (512.26 ± 110.58 pg/ml vs. 388.56 ± 123.67 pg/ml, *p* < 0.05, Figure 1B, Table 1).

### 3.2 Correlation between CR‐1 and VEGF levels in NSCLC patients

There is a significant positive correlation between CR‐1 and VEGF levels in NSCLC patients (r = 0.518, *p* < 0.01).

### 3.3 Comparison of serum CR‐1 and VEGF levels depending on clinical characteristics in NSCLC patients

The levels of serum CR‐1 were higher in stage IV than in stage I ~ III (*p* < 0.01). In addition, the levels of serum VEGF increased with staging, and the serum VEGF levels in stage IV NSCLC were higher than those of early NSCLC (*p* < 0.01, Table 2).

**TABLE 2:** Comparison of serum CR‐1 and VEGF levels depending on clinical characteristics in NSCLC patients

Variables	Cases, No.	CR‐1 (ng/ml)	*p*	VEGF (pg/ml)	*p*
Age (years)			0.814		0.217
≥60	200	4.12 ± 1.53		514.08 ± 102.66	
<60	112	4.26 ± 1.51		508.36 ± 107.18	
Gender			0.337		0.678
Male	140	3.56 ± 1.76		531.27 ± 103.56	
Female	172	4.18 ± 1.65		518.33 ± 105.85	
Histology			0.268		0.358
ADC	218	4.35 ± 1.57		528.18 ± 110.56	
SCC	94	3.77 ± 1.38		549.39 ± 108.49	
Differentiation			0.267		0.536
Well‐moderate	208	4.18 ± 1.93		546.02 ± 105.18	
Poor	104	4.17 ± 1.08		539.78 ± 118.35	
TNM stage			0.001		0.051
I	40	2.14 ± 1.36		389.46 ± 104.32	
II	36	3.28 ± 1.05		493.42 ± 112.14	
IIIA	60	4.25 ± 1.21		504.15 ± 104.36	
IIIB	30	4.58 ± 1.36		526.36 ± 109.67	
IIIC	44	4.89 ± 1.54		535.59 ± 118.78	
IV	102	5.18 ± 1.13		542.76 ± 121.12	
T status			0.001		0.002
T1	48	3.24 ± 1.26		451.57 ± 103.68	
T2	58	3.77 ± 1.56		522.46 ± 118.43	
T3	84	4.38 ± 1.08		533.09 ± 103.87	
T4	122	4.63 ± 1.67		558.27 ± 106.57	
Lymph node metastasis			0.001		0.001
Absent	116	1.33 ± 0.61		388.26 ± 107.88	
Present	196	4.38 ± 1.54		513.46 ± 124.43	
Distant metastasis			0.001		0.001
Absent	210	2.35 ± 1.38		414.35 ± 113.63	
Present	102	5.18 ± 1.13		542.76 ± 121.12	

Abbreviations: ADC, Adenocarcinoma; SCC, Squamous cell carcinoma.

The VEGF levels in stage I did not differ from those in the controls, but serum CR‐1 levels in stage I were higher than those of the controls (*p* < 0.05). The CR‐1 and VEGF levels were closely related to lymph node metastasis and distant metastasis (*p* < 0.05).

There were no significant correlation between CR‐1 and VEGF expression and age, gender, histological type, and differentiation (Table 2).

### 3.4 Diagnostic value of CR‐1 and VEGF levels in NSCLC patients

**FIGURE 2:**
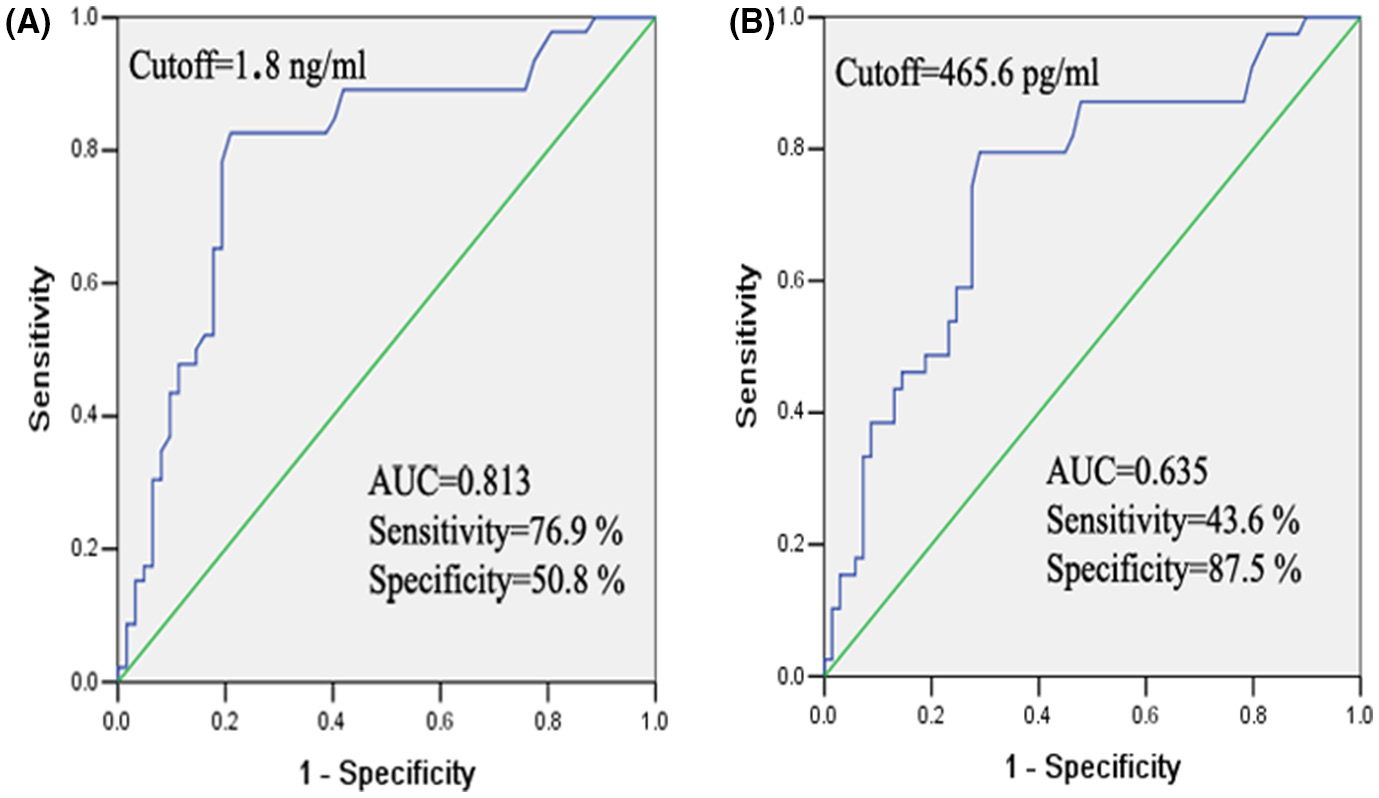
ROC curves for the serum CR‐1 (A) and VEGF (B) in differentiating the lung cancer and control groups

The receiver operating characteristic (ROC) curve of CR‐1 and VEGF level were established to identify the cutoff values. The sensitivity, the specificity of CR‐1 was 76.9% and 50.8% in NSCLC patients with a cutoff value of 1.8 ng/mL. With a cutoff value of 465.6 pg/ml, the sensitivity, the specificity of VEGF levels was 43.6% and 87.5%, respectively. The expression level of the two proteins was compared in terms of area under the curve (AUC), and the AUC of CR‐1 was larger than VEGF **(**Figure 2).

### 3.5 Relationship between CR‐1 levels and the recurrence in NSCLC patients

The levels of serum CR‐1 in patients with postoperative recurrence were higher than those of patients without recurrence in NSCLC patients with complete surgical resection (Table 3). On the contrary, there was no difference in VEGF levels for tumor recurrence.

**TABLE 3:** Relationship between serum CR‐1 and VEGF levels and the recurrence in NSCLC patients

Recurrence	Cases, No.	CR‐1 (ng/ml)	*p*	VEGF (pg/ml)	*p*
Recurrence within 1‐year in operated NSCLC (stage ≤ III A)			0.015		0.776
Yes	36	3.54 ± 1.16		506 ± 108.77	
No	100	2.14 ± 1.02		498 ± 118.53	
Recurrence within 2‐year in operated NSCLC (stage ≤ III A)			0.012		0.817
Yes	60	4.13 ± 1.21		514 ± 112.38	
No	76	2.01 ± 1.34		499 ± 109.78	
Recurrence within 2‐year in operated NSCLC (stage ≤ II)			0.036		0.513
Yes	20	3.67 ± 1.55		498 ± 103.53	
No	56	1.56 ± 1.38		472 ± 112.45	

### 3.6 Correlation of serum CR‐1 and VEGF levels with overall survival

**FIGURE 3:**
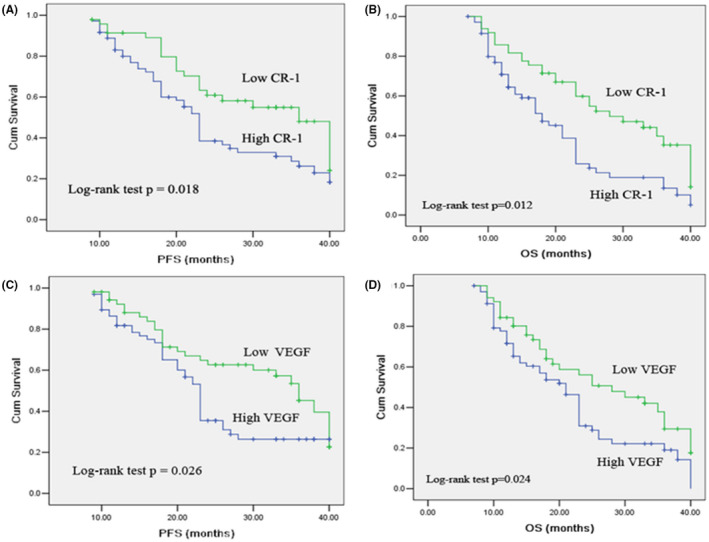
Kaplan–Meier survival curves for PFS and OS in patients according to the serum CR‐1 (A and B) and VEGF (C and D) levels

The impact of serum CR‐1 concentration on lung cancer survival was also examined with the Cox proportional hazards regression model. The results of univariate and multivariate analysis are presented in Table 4. Univariate analysis showed that serum CR‐1 and VEGF levels were significantly correlated with PFS and OS. In multivariate analysis, high CR‐1 (≥1.8 ng/ml) and high VEGF (≥ 465.6 pg/ml) was found to be significantly associated with a shorter PFS and OS. Kaplan–Meier survival curves further demonstrate that lung cancer patients with high CR‐1 and high VEGF have substantially shorter PFS and OS, compared to those with low CR‐1 and low VEGF patients (Figure 3A–D). As expected, TNM stage and distant metastases were found to be strongly associated with decreased PFS and OS, in both univariate and multivariate analyses.

**TABLE 4:** Univariate and multivariate Cox analysis of variables for PFS and OS in NSCLC

Variables	PFS			OS		
	HR	95% CI	*p*	HR	95% CI	*p*
Univariate analysis	0.67	0.35–1.32	0.238	1.36	0.12–4.35	0.734
Age (≥ 60 vs. < 60)	1.15	0.69–1.90	0.579	1.42	0.81–2.48	0.218
Gender (male vs. female)	2.18	0.82–5.77	0.118	1.13	0.53–2.15	0.701
Histology (ADC vs. SCC)	1.52	0.33–7.07	0.593	1.33	0.65–2.76	0.438
Differentiation (well‐moderate vs. poor)	1.12	0.67–1.85	0.689	1.52	0.33–7.07	0.593
TNM stage (I + II vs. III + IV)	2.91	2.02–4.20	0.001	2.94	1.31–4.52	0.002
Lymph node metastases(positive vs. negative)	2.13	1.15–3.94	0.016	1.73	1.08–2.79	0.024
Distant metastases (positive vs. negative)	2.10	0.97–3.53	0.029	1.99	0.99–2.71	0.044
VEGF (high vs. low)	1.86	1.49–2.31	0.001	2.93	1.77–4.87	0.001
CR‐1 (high vs. low)	1.71	1.10–2.65	0.017	1.82	0.91–3.63	0.024
Multivariate analysis						
Age (≥ 60 vs. < 60)	1.98	0.78–5.02	0.152	1.65	0.74–3.66	0.220
Gender (male vs. famale)	1.36	0.93–1.99	0.113	1.52	0.97–2.11	0.098
Histology (ADC vs. SCC)	1.28	0.88–1.87	0.196	1.06	0.80–1.40	0.061
Differentiation (well‐moderate vs. poor)	1.10	0.42–2.89	0.848	0.72	0.39–1.35	0.305
TNM stage (I + II vs. III + IV)	2.48	1.36–4.50	0.003	2.18	1.09–4.35	0.026
Lymph node metastases (positive vs. negative)	1.96	1.16–3.32	0.013	1.32	0.84–2.07	0.233
Distant metastases (positive vs. negative)	2.65	1.65–4.76	0.007	2.94	1.31–4.52	0.002
VEGF (high vs. low)	1.02	1.01–1.03	0.002	1.56	1.13–2.14	0.002
CR‐1 (high vs. low)	1.75	1.34–2.29	0.001	2.23	1.29–3.86	0.004

Abbreviations: ADC, Adenocarcinoma; CI, Confidence interval.; HR, Hazard ratio; OS, Overall survival; PFS, Progression‐free survival; SCC, Squamous cell carcinoma.

## 4 DISCUSSION

CR‐1 promotes proliferation, survival, migration, and invasion of tumor cells and tumor angiogenesis.^14^ High expression of VEGF in lung cancer is associated with prognosis.^15^, ^16^ Elevated serum VEGF levels in patients with lung cancer are related to tumor progression and prognosis.^17^, ^18^, ^19^

Previous studies have shown that overexpression of CR‐1 in lung cancer is associated with poor prognosis. However, few studies have assessed the clinical significance of serum CR‐1 levels in lung cancer. Our results suggest that CR‐1 was superior to VEGF in the differential diagnosis of NSCLC, especially in patients with and without distant metastasis.

In this study, serum VEGF levels were significantly associated with the stage of NSCLC and positively correlated with serum CR‐1 levels. Previous studies have explored the relationship between serum VEGF levels and stage in patients with lung cancer, but the results are inconsistent. Laack et al. reported that there was a significant positive relationship between serum VEGF and clinical stage.^**2**0^ Our results are basically in line with theirs.

The increase of serum CR‐1 levels in patients with NSCLC depends on the clinical stage. The significant increase of serum CR‐1 level in patients with distant metastasis theoretically indicates that angiogenesis is a necessary condition for tumor progression. The relationship between angiogenesis and systemic metastasis has been confirmed in various tumors, including lung cancer.^**2**1^

Previous studies have shown that CR‐1 expression is related to tumor prognosis.^6^, ^22^ Xu et al. have shown that the increased expression of CR‐1 mRNA is closely related to the poor prognosis of patients with NSCLC.^10^ This study supports the results of more and more literatures that the expression of CR‐1 is related to the prognosis of cancer. We observed a significant correlation between elevated serum CR‐1 levels and poor prognosis in patients with NSCLC. In addition, the results suggest that serum CR‐1 was a marker of tumor recurrence.

Although serum CR‐1 is a potentially useful auxiliary diagnostic or prognostic marker, it is insufficient as a single diagnostic marker for lung cancer. Although the difference of CR‐1 between lung cancer patients and controls was more significant than that of VEGF, the considerable overlaps and numerical discrepancy between the two groups weakened its practicability as a clinical marker, and ROC analysis showed that it did not have a strong ability to distinguish. More large‐scale prospective studies are warranted to confirm the findings.

In a word, our results suggest that serum CR‐1 and VEGF may be a potential diagnostic and prognostic markers for NSCLC.

## CONFLICT OF INTEREST

The authors declare no any conflict of interest in this work.

## AUTHOR CONTRIBUTIONS

CHX carried out most of the experiment and wrote this manuscript; LL and YCW did the ELISA; JZ and WW collected data; WW helped with the design and all through the research. All authors read and approved the final manuscript.

## ETHICS APPROVAL AND CONSENT TO PARTICIPATE

This study was approved by the Ethics Committee of Nanjing Chest Hospital and was carried out in accordance with national law and the current revised Declaration of Helsinki. Informed consent was obtained from all participants in the study.
